# Comparative Effect of Water‐Diluted Stool on the Growth and Development of *Rhabditis* sp., *Strongyloides stercoralis*, and Hookworm

**DOI:** 10.1155/vmi/3238555

**Published:** 2026-10-07

**Authors:** Attarat Pattanawongsa, Patthanasak Khammaneechan, Blego Sedionoto, Prasit Na-ek, Witthaya Anamnart

**Affiliations:** ^1^ School of Pharmacy Walailak University Nakhon Si Thammarat 80161 Thailand wu.ac.th; ^2^ Drug and Cosmetics Excellence Center Walailak University Nakhon Si Thammarat 80161 Thailand wu.ac.th; ^3^ School of Public Health Walailak University Nakhon Si Thammarat 80161 Thailand wu.ac.th; ^4^ EC for PHR Walailak University Nakhon Si Thammarat 80161 Thailand wu.ac.th; ^5^ Department of Environmental Health Faculty of Public Health Mulawarman University Samarinda East Kalimantan Indonesia unmul.ac.id; ^6^ Department of Medical Science School of Medicine Walailak University Nakhon Si Thammarat 80161 Thailand wu.ac.th; ^7^ Research Center in Tropical Pathobiology Walailak University Nakhon Si Thammarat 80161 Thailand wu.ac.th; ^8^ School of Allied Health Sciences Walailak University Nakhon Si Thammarat 80161 Thailand wu.ac.th

**Keywords:** drought, El Niño, La Niña, *Rhabditis*sp

## Abstract

**Background:**

Raw vegetables in southern Thailand frequently carry *Rhabditis* sp. Epidemiological shifts (2008–2023) show an increase in *Rhabditis* sp. colonization alongside declining hookworm and *Strongyloides stercoralis* infections, suggesting a potential association with climate variations. To evaluate moisture effects under controlled conditions, we compared nematode egg and first‐stage larval development using an agar plate culture (APC) control and a 1:20 water‐diluted stool suspension (SS) simulating wet environments.

**Methods:**

Stool samples positive for *Rhabditis* sp. from 18 humans (12 co‐infected with hookworm or *S. stercoralis*) and 25 dogs (10 co‐infected with hookworm) were cultured concurrently in APC and SS. Following a 6‐day incubation at room temperature, we quantified *Rhabditis* sp. developmental stages and third‐stage larvae (L3) of hookworm and *S. stercoralis*.

**Results:**

In APC, all human (18/18) and dog (25/25) cases achieved complete *Rhabditis* sp. development, supporting significantly more developmental stages than SS. In SS, only 22.2% of human and 28.0% of dog cases exhibited partial development, confirming that water dilution impairs *Rhabditis* sp. growth. Conversely, hookworms reached the L3 stage in all human (8/8) and dog (10/10) cases across both methods without significant differences. For *S. stercoralis* (7/7 humans), both methods yielded L3, though APC provided higher yields.

**Conclusions:**

Our experimental findings demonstrate that *Rhabditis* sp. development is significantly more vulnerable to water dilution than hookworm or *S. stercoralis*. While this impairment is hypothesized to stem from reduced bacterial abundance, this mechanism remains speculative and requires direct microbiological confirmation. These laboratory observations suggest that lower‐moisture environments may favor *Rhabditis* sp. developmental success over other nematodes, though broader ecological and epidemiological implications require further study given the limited sample size.

## 1. Introduction

Free‐living nematodes, predominantly *Rhabditis* sp., are frequently recovered during the diagnosis of strongyloidiasis and hookworm infections when using agar plate culture (APC). This co‐occurrence can lead to significant clinical misdiagnosis [1, 2]. For instance, eggs of free‐living nematodes in stool matrices may be misidentified as hookworm eggs under the Kato–Katz technique or direct fecal smears. Similarly, first‐stage larvae (L1) can be mistaken for *Strongyloides stercoralis* larvae during direct fecal smears, formalin‐ether concentration, or Baermann examinations, while adult free‐living worms may be misclassified as *S. stercoralis* adults on APC [2, 3]. Although *Rhabditis* sp. has been documented as a causative agent of otitis in animals [4], ear infections in humans [5], and diarrhea in children [6], documented cases of definitive intestinal rhabditid infections remain rare [7]. While the precise route of transmission remains unclear, *Rhabditis* sp. is hypothesized to enter the human digestive tract via the consumption of contaminated raw vegetables. Surveys from southern Thailand have demonstrated a remarkably high prevalence (81%–100%) of free‐living nematodes, primarily *Rhabditis* sp., on raw produce [8, 9]. Given that raw vegetable consumption is a common dietary habit in this region [10], ingestion of these nematodes may occur frequently; however, such cases are usually asymptomatic and likely represent transient gut colonization rather than true active infection.

The primary study region is Nakhon Si Thammarat, located in southern Thailand, which features a tropical rainforest climate characterized by a prolonged 9–10‐month rainy season and a short 2–3‐month dry season. Historically (2006–2013), the area maintained an average annual rainfall of 2916 mm distributed across approximately 174 rainy days (Table S1). However, between 2014 and 2023, the region experienced climatic variations consisting of three El Niño events (2014–2015, 2019–2020, 2022–2023) and two La Niña events (2017–2018, 2021–2022), which introduced notable periods of drought and dry spells (Tables S2–S5). During these El Niño years, the regional average maximum temperature reached 35.0°C–35.3°C (with peaks up to 38.4°C), and relative humidity fell compared to La Niña periods (Tables S6 and S7).

Following these major climate cycles, a general decline in annual rainfall and rainy days was noted (Tables S2 and S3). Interestingly, epidemiological data from these periods revealed an inverse trend between conventional soil‐transmitted helminths and rhabditid nematodes. During the high‐rainfall period of 2008–2012, recorded rates of *Rhabditis* sp. colonization, hookworm infection, and strongyloidiasis were 0.08%, 61%, and 30%, respectively. Conversely, during the lower‐rainfall period spanning 2016–2023, *Rhabditis* sp. detection rose to 2.22%, while hookworm and strongyloidiasis rates decreased to 13.6% and 10.7%, respectively (Table S8).

While these field observations are strictly ecological and observational, they suggest a potential association between climate‐driven moisture variations and nematode prevalence. It is possible that frequent or excessive rainfall patterns create a less favorable matrix for *Rhabditis* sp. while benefiting hookworm and *S. stercoralis* cycles. Conversely, periods of reduced moisture or drought may exhibit the opposite trend. In a preliminary laboratory experiment, we compared the growth of *Rhabditis* sp. from five feline stool samples using both an APC control and a 1:20 water‐diluted stool suspension (SS). *Rhabditis* sp. failed to develop or exhibited severely restricted growth (recovering only ∼10% of the yield found in APC) in the fluid medium, whereas *Strongyloides* sp. maintained high developmental viability (∼70%) under identical dilution conditions.

To systematically test the hypothesis that fluid saturation differentially affects these species, this study compared the egg and first‐stage larval development of *Rhabditis* sp., hookworm, and *S. stercoralis* under controlled laboratory conditions. We utilized a 1:20 water‐diluted SS to simulate high‐moisture environmental conditions, using the standard APC as an optimized control environment.

## 2. Materials and Methods

### 2.1. Study Areas

We studied humans at Villages 11 and 13 in the Mokkhalan subdistrict (Figure 1, bottom right), located 8 km from our lab at Walailak University. We sampled dogs at Walailak University and in nearby Thaiburi and Thasala subdistricts (Figure 1, top right). All locations are in the Thasala district, Nakhon Si Thammarat, southern Thailand. This area is on the peninsula between the Pacific and Indian Oceans (Figure 1).

**FIGURE 1:**
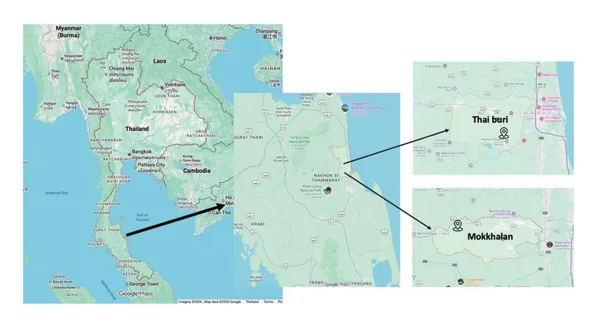
The maps show two study areas: Mokkhalan subdistrict (bottom‐right) and Thai buri subdistrict (top‐right), Thasala district, Nakhon Si Thammarat, southern Thailand. The maps were captured from Google Maps.

### 2.2. Study Design

We conducted a descriptive comparative study in humans (2016–2023) and dogs (2020). Human stools were collected in the morning and examined within 4 h. Triplicate APC detected *Rhabditis* sp., *S. stercoralis*, and hookworm. We used triplicate APC from a single sample instead of collecting stools for 3 days. Participants positive for *Rhabditis* sp. or co‐infected with hookworm received a single 400‐mg oral dose of albendazole. Those with *S. stercoralis* co‐infection received 400‐mg albendazole daily for 3 days. We assessed treatment outcomes by triplicate APC after 3 weeks. Before treatment, 18 *Rhabditis* sp.‐positive stools were collected 7 days after the first sample and cultured using a 1:20‐water‐diluted SS and APC. Six were positive for *Rhabditis* sp. alone. Five had *Rhabditis* sp. with hookworm, four had *S. stercoralis*, and three had both hookworm and *S. stercoralis*. We evaluated stray dogs in a similar way in 2020. Of 25 *Rhabditis* sp.‐positive dog stools, 15 were *Rhabditis* sp. alone and 10 were co‐infected with hookworm. After the dogs had defecated, we transferred the dog stools to containers and processed them within 4 h. Both human and dog stools were cultured using APC and SS for comparison. Stool consistency was normal: formed for dogs, soft for humans.

### 2.3. Laboratory Procedure

We used triplicate SS and APC to compare growth and development among the three worms. We also observed the daily growth and development of the three worms in both APC and SS. SS [11] was processed as follows. One gram of stool was diluted with 19 mL of DW in a 150‐mm dish. This formed a 2‐mm suspension. The plate was incubated at room temperature (26°C–33°C) for 6 days. To count the growing stages of *Rhabditis* sp., L3 of *S. stercoralis*, and hookworm, we used several steps. First, we placed the culture dish on a stereo microscope stage for 5 min. *S. stercoralis* L3, exposed to microscope light, swam to the water surface. We transferred these L3 to a 150‐mm dish using a 20‐μL automatic pipette under a 7.5x magnification stereo microscope. Next, we killed the L3 with 10 mL of 10% formalin and counted them at 7.5x magnification. Then, we added 10 mL of 10% formalin to the remaining SS. After 10 min, we counted the number of dead growing stages of *Rhabditis* sp. (excluding free‐living adults) in one of 36 grids of the dish and multiplied the count by 36. Finally, we counted the motile hookworm L3. We reported the number of L3 or growing stages per gram of stool.

APC [12] was processed as follows. We placed 1 g of stool at the center of the nutrient agar surface. The plate was incubated at room temperature for 6 days. We then counted the growth stages of *Rhabditis* sp., *S. stercoralis* L3, and hookworm L3 as follows. First, we added 10 mL of DW onto the agar surface in the 90‐mm plate on a stereo microscope stage. After 5 min, *S. stercoralis* L3 swam to the water surface. We transferred these L3 to a 150‐mm dish using a 20‐μL automatic pipette under a 7.5x stereo microscope. We repeated this step for the remaining *S. stercoralis* L3. The L3 were fixed in 10 mL of 10% formalin and counted at 7.5x magnification. Some L3 were identified with a compound microscope at 40x magnification. Next, we added 10 mL of 10% formalin to the agar surface. After 10 min, the flushed, formalin‐containing worms were transferred back to a 150‐mm dish. We repeated the formalin addition and transfer for any remaining worms. *Rhabditis* sp. growing stages were counted in one of 36 grids of the 150‐mm dish and multiplied by 36. We then counted the motile hookworm L3. We reported the number of L3 or growing stages per gram of stool.

Notably, all stages of *Rhabditis* sp. and *S. stercoralis* died within 7 min after adding 10% formalin. The L3 of hookworm died after 30 min or more (Table S9). Possibly, hookworm L3 is a nonfeeding form. When *S. stercoralis* L3 are exposed to microscope light, all of them will swim to the water surface [13].

In addition, *S. stercoralis* L3 has a slim body with a long, filariform esophagus comprising 1/3–1/2 of the body length and a notched tail. In contrast, hookworm has a fat body compared to *S. stercoralis* L3, a short filariform esophagus, and a pointed tail. Free‐living males and females of *Rhabditis* sp. generally resemble those of *S. stercoralis*, except for the long buccal cavity.

### 2.4. Statistical Analyses

All data and statistical analyses were performed using IBM SPSS Statistics (version 29.0.0) (New York, USA). Due to the small sample sizes, the median and interquartile range were calculated for all data in the present study. Fisher’s exact test was used to assess differences in the numbers of *Rhabditis* sp., hookworm, and *S. stercoralis*‐positive dog and human samples between APC and SS. The Mann–Whitney *U* test was used to analyze differences in the number of growing stages of *Rhabditis* sp., L3 of *S. stercoralis,* and hookworm between APC and SS in humans and dogs. A two‐sided *p* value of < 0.05 was considered significant.

## 3. Results

### 3.1. Evidence of *Rhabditis* sp. Gut Colonization

*Rhabditis* sp. positive human cases likely represent true intestinal colonization rather than transient environmental contamination. This is supported by the fact that *Rhabditis* sp. successfully grew and developed on triplicate APC plates. Furthermore, a second stool collection obtained 7 days after the baseline sample from these *Rhabditis* sp.‐positive individuals remained persistently positive. At 21 days post‐treatment, no *Rhabditis* sp. growth was observed on APC, indicating that these rhabditid worms are highly sensitive to treatment with either a single dose or three consecutive daily doses of 400 mg albendazole (Table S10).

### 3.2. Nematode Behavioral Observations

Twenty‐four hours after incubation, some hatched hookworm L1 larvae crawled out of the stool mass briefly before returning. After a few days, these L1 larvae either transitioned into a resting phase within the agar matrix or remained nonmotile within the SS medium. For *S. stercoralis*, L1 larvae developed into either infective L3 larvae or free‐living adult males and females within approximately 24 h in both APC and SS conditions. In APC, these adult worms subsequently emerged on the agar surface adjacent to the stool mass. Approximately 18–24 h post‐mating, free‐living females deposited embryonated eggs that hatched immediately, with the resulting L1 larvae developing into infective L3 within 24 h. For *Rhabditis* sp., a few days post‐cultivation, adult males and females were observed crawling and mating on the agar surface, producing numerous sequential developmental stages. The majority of these life stages were concentrated on the agar surface in areas covered by a dense, swarming bacterial film.

### 3.3. Comparative Growth Success in SS Versus APC

A primary finding was that only 22.2% (4/18) of *Rhabditis* sp.‐positive human stool samples exhibited growth in SS compared to 100% (18/18) in APC (*p* < 0.001, OR = 119.2; 95% CI: 5.9, 2398.4). Subgroup analyses revealed identical trends, showing significantly lower growth success in SS for *Rhabditis* sp. mono‐cultures and *Rhabditis* sp. co‐cultures with hookworm compared to APC. However, for subgroups containing *Rhabditis* sp. co‐cultured with *S. stercoralis*, or co‐cultured with both hookworm and *S. stercoralis*, statistically significant differences in growth success were not observed, which is likely attributable to the limited sample sizes within these specific cohorts. In stark contrast, both hookworm (8/8) and *S. stercoralis* (7/7) achieved 100% growth success in both SS and APC media (Figure 2 and Table S11).

**FIGURE 2:**
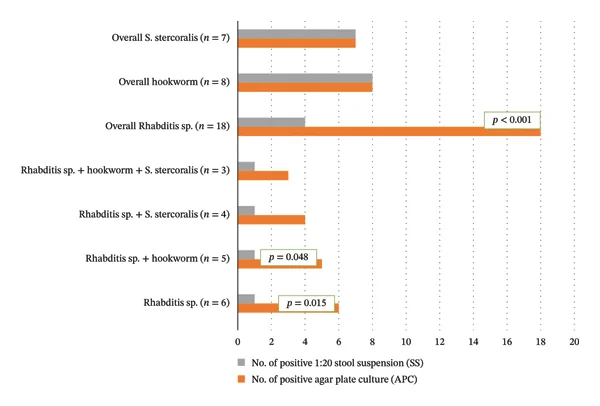
Comparative numbers of hookworm‐, *S. stercoralis*‐, and *Rhabditis* sp. mono‐cultured‐positive and co‐cultured‐positive human cases with hookworm and/or *Strongyloides stercoralis* between agar plate culture and 1:20 stool suspension.

In canine specimens, a principal finding was that only 28.0% (7/25) of *Rhabditis* sp.‐infected stools demonstrated growth in SS, compared to 100% (25/25) in APC (*p* < 0.001, OR = 125.8; 95% CI: 6.8, 2343.4). Conversely, hookworm infections (10/10) consistently achieved 100% growth success across both culture methods (Figure 3 and Table S12).

**FIGURE 3:**
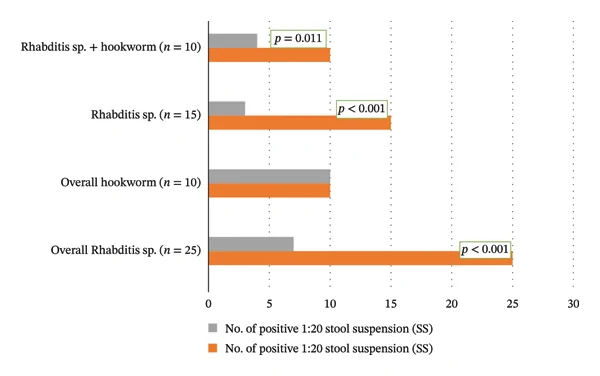
Comparative numbers of hookworm‐ and *Rhabditis* sp. mono‐cultured‐positive and co‐cultured‐positive dog stool cases with hookworm between agar plate culture and 1:20 stool suspension.

### 3.4. Quantitative Effects of Stool Dilution on Larval Yields

For human specimens, the primary findings indicated that all *Rhabditis* sp.‐positive subgroups produced significantly fewer developmental stages in SS than in APC. In contrast, the absolute numbers of hookworm L3 larvae did not differ significantly between SS and APC. However, the yields of *S. stercoralis* L3 larvae were significantly lower in SS than in APC within co‐cultured samples (Figure 4 and Table S13).

**FIGURE 4:**
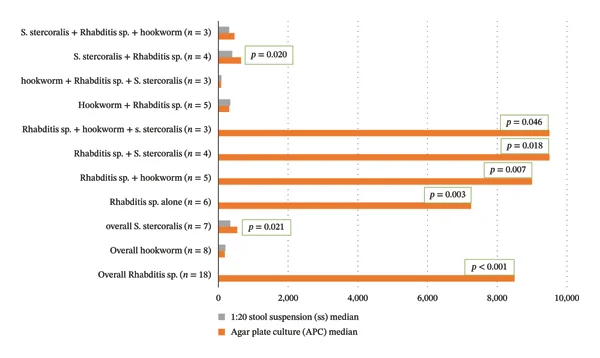
Comparative median number of *Rhabditis* sp. growing stages and third‐stage larvae of hookworm and *Strongyloides stercoralis* per gram of human stools between agar plate culture and 1:20 stool suspension.

In dog samples, the absolute number of *Rhabditis* sp. developmental stages was consistently lower in SS than in APC across all examined groups, whereas hookworm L3 yields remained statistically similar under both culture conditions (Figure 5 and Table S14).

**FIGURE 5:**
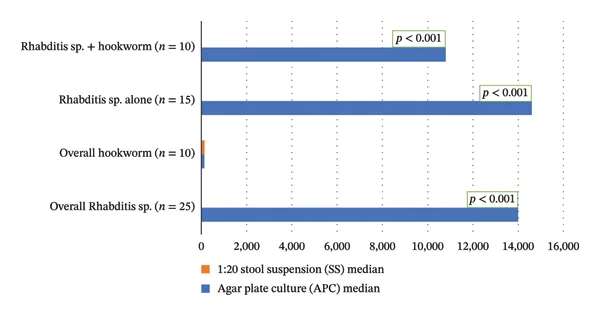
Comparative median number of hookworm third‐stage larvae, and median number of rhabditid growing stages/gram of dog stools between agar plate culture and 1:20 stool suspension.

## 4. Summary of Experimental Findings

In summary, these laboratory data indicate that liquid dilution suppresses the growth and development of *Rhabditis* sp. to a significantly greater extent than that of *S. stercoralis* or hookworm. These findings demonstrate that *Rhabditis* sp. is highly susceptible to wet or highly diluted fluid environments in vitro, suggesting that lower‐moisture matrices may optimize its developmental survival relative to the other two nematode species.

## 5. Discussion

Bacteria in undiluted stool placed on nutrient agar (APC) grow within the stool mass and on the adjacent agar surface, forming visible colonies within 24 h. Motile larvae may facilitate the mechanical dispersal of these bacteria across the plate, mimicking a bacterial streaking effect. In contrast, inside a 1:20 water‐diluted SS, the relative concentration of nutrients and baseline bacteria is fundamentally reduced. Because no exogenous nutrients are introduced to support microbial proliferation in the liquid phase, bacterial abundance in SS is hypothesized to be lower than that on APC, preventing visible colony formation. However, because bacterial density and colony‐forming units (CFUs) were not explicitly quantified in this study, this microbiological dynamic remains inferential and warrants direct experimental verification.

Hookworm L1 may require organic substances from bacteria, microorganisms, stool, or soil [13, 14]. *S. stercoralis* L1 may require bacteria and soluble stool components [11, 13]. *Rhabditis* sp. may require bacteria or other microorganisms for growth, but not soluble stool components. For example, *Caenorhabditis elegans*, a type of *Rhabditis* sp., develops in an agar‐based medium with a bacterial lawn. *C. elegans* also grows in a liquid medium with concentrated *E. coli* as food and is placed on a shaker [15].

Hookworm L1 feeds on organic substances in undiluted stool placed on nutrient agar. This results in full growth and development. When the stool is diluted, abundance decreases. Hookworm L1 still feeds on bacteria in the diluted stool and may get extra organic substances. As a result, growth and development are not affected in SS. L1 growth and development are affected only if the stool is diluted to the level of DW due to a lack of organic matter. If bacteria are added, L1 growth and development continue, and L1 becomes L3 [13].1

*S. stercoralis* L1 develops into free‐living males and females, which mate and produce fewer L3 in SS compared to APC. This is because *S. stercoralis* L1 requires both bacteria and soluble stool components for growth [11, 13]. Feeding L1 on bacteria and stool components in undiluted stool on nutrient agar may lead to maximum L3 production. In SS, L1 may obtain sufficient bacteria but insufficient soluble stool components, resulting in fewer L3. Dilution affects L1 growth and development. More dilution leads to less L3 production. When stool is diluted more than 1:160, L1 fails to develop into free‐living adults [11]. Even if bacteria are added, L3 cannot be produced [13]. Thus, 1:20‐diluted stool is suitable to compare *S. stercoralis* L3 production and other developmental stages with those of the other two worms.

*Rhabditis* sp. exhibited complete development on APC but experienced profound developmental failure in SS. On APC, hatching L1 larvae were observed feeding on ambient bacteria within the stool and migrating outward to graze on swarming bacterial lawns, eventually progressing to free‐living adults within 48–60 h. Given that rhabditid nematodes depend heavily on high‐density bacterial feeding [15], we hypothesize that the water dilution in SS reduced the available bacterial biomass below a critical threshold required for their development. However, because direct microbial counts were not performed, this mechanistic explanation remains speculative.

In natural settings, *Rhabditis* sp. typically inhabits soil or decaying plant debris, feeding on rapid‐growth environmental bacteria [16]. Our laboratory findings showed that water dilution severely limits *Rhabditis* sp. development compared to APC. On an ecological scale, this observed susceptibility to liquid dilution shares a temporal parallel with local climate observations. Between 2014 and 2023, southern Thailand experienced climatic variations—including El Niño and La Niña cycles—characterized by prolonged dry seasons and reduced annual rainfall days. While a direct causal link between climate variation and parasite prevalence cannot be definitively established from these observational data, it is plausible that lower ambient moisture periods correlate with reduced environmental stool dilution, potentially creating conditions that favor rhabditid developmental success.

Among the three investigated nematodes, hookworm demonstrated the highest developmental resilience in 1:20 water‐diluted stool, followed by *S. stercoralis*, while *Rhabditis* sp. was the most severely impaired. While these differences highlight varying tolerances to liquid environments in vitro, they do not directly measure desiccation tolerance. Nonetheless, literature suggests that while hookworms and *S. stercoralis* are highly sensitive to drying [14, 17], certain rhabditid species possess superior desiccation survival mechanisms; for instance, significant proportions of *Rhabditis blumi* and *Rhabditis rainai* can tolerate low relative humidity states [18].

Interestingly, the local prevalence of other soil‐transmitted helminths, such as *Ascaris lumbricoides* and *Trichuris trichiura*, declined following regional dry spells [3]. Because these helminths infect hosts via egg ingestion, their decline under dry conditions contrasts with the rising trends observed for *Rhabditis* sp. colonization. Given our small sample size, broad epidemiological inferences regarding these opposing trends must be made with caution. However, the persistence of *Rhabditis* sp. may be linked to alternative environmental reservoirs. Rhabditid larvae are frequent contaminants of fresh vegetables commonly consumed raw in local Thai diets [10], with some surveys reporting free‐living stages of *Rhabditis* sp. in 80%–100% of tested vegetable samples [8, 9]. The agricultural practice of using organic plant fertilizers, which are rich in proliferating bacteria, may provide a highly favorable microenvironment that supports *Rhabditis* sp. growth prior to human contact, though this pathway requires further field validation.

## 6. Limitations

The primary limitation of this study is the small sample size (*n* = 18 for human *Rhabditis*‐positive samples), which substantially limits the statistical power of subgroup analyses where observations ranged between 3 and 6 samples, resulting in wide confidence intervals. Consequently, the findings presented here should not be used to make broad ecological or epidemiological generalizations. Furthermore, bacterial abundance and specific CFUs were not measured or compared between the APC and SS groups. The proposed role of bacterial density in driving the developmental differences remains a conceptual hypothesis that requires direct microbiological validation in future studies.

## 7. Conclusions

Our experimental laboratory findings demonstrate that *Rhabditis* sp. development is highly vulnerable to water dilution compared to hookworm and *S. stercoralis*. In vitro, a 1:20 water dilution of stool significantly inhibits *Rhabditis* sp. growth, a mechanism hypothetically attributed to a reduction in bacterial abundance that requires direct experimental confirmation. Conversely, hookworm and *S. stercoralis* exhibit greater developmental resilience under diluted liquid conditions. While these observational data show a temporal parallel between regional climate shifts (such as El Niño and La Niña dry periods) and increased *Rhabditis* sp. colonization, a direct causal relationship cannot be concluded. These laboratory observations primarily indicate that lower‐moisture environments may optimize the developmental success of *Rhabditis* sp. relative to other nematodes by minimizing matrix dilution, though the broader epidemiological and clinical implications of these findings require further investigation using larger sample sizes.

## Author Contributions

Attarat Pattanawongsa: conceptualization, methodology, investigation, and writing–review and editing. Patthanasak Khammaneechan: conceptualization, investigation, data curation, and writing–review and editing. Blego Sedionoto: investigation, data curation, and writing–review and editing. Prasit Na‐ek: methodology and writing–review and editing. Witthaya Anamnart: conceptualization, supervision, and writing–original draft.

## Funding

No funding was received for this manuscript.

## Ethics Statement

This study received approval from the Ethics Committee in Human Research at Walailak University, Thailand, under approval numbers 15/071, 20‐232‐01 and 23/028‐01. Participants reviewed the participant information sheet and provided their written informed consent to participate in the study. For participants under 18, their guardians obtained written consent. The research on stray dogs may not require the approval from animal ethics committee because we did not handle or monitor stray dogs. After the dog had already defecated, we transferred each dog’s stool from cement or soil surfaces to plastic containers.

## Consent

The authors have nothing to report.

## Conflicts of Interest

The authors declare no conflicts of interest.

## Supporting Information

Additional supporting information can be found online in the Supporting Information section.

## Supporting information


**Supporting Information 1** TableS1 ‐ Supporting information 1 Table. Rainfall and rainy days in Nakhon Si Thammarat during 2006–2013.

**Supporting Information 2** TableS2 ‐ Supporting information 2 Table. Rainfall and number of rainy days measured by the local meteorological station 0064.

**Supporting Information 3** TableS3 ‐ Supporting information 3 Table. Rainfall and number of rainy days measured by the local meteorological station 0747.

**Supporting Information 4** TableS4 ‐ Supporting information 4 Table. Rainfall and rainy days in Nakhon Si Thammarat during 2013–2016.

**Supporting Information 5** TableS5 ‐ Supporting information 5 Table. Rainfall in mm in Thailand, the eastern coast of southern Thailand, Nakhon Si Thammarat (NST) Province, and 2 local meteorological stations, and dry spell length (DSL) days in NST Province during 2014–2023.

**Supporting Information 6** TableS6 ‐ Supporting information 6 Table. Maximum temperature in Nakhon Si Thammarat during 2013–2023.

**Supporting Information 7** TableS7 ‐ Supporting information 7 Table. Relative humidity in Nakhon Si Thammarat during 2013–2023.

**Supporting Information 8** TableS8 ‐ Supporting information 8 Table. Comparing the prevalence of hookworm infection and strongyloidiasis with *Rhabditis* sp. colonization rate before and after the 2014–2016 El Niño‐induced drought.

**Supporting Information 9** TableS9 ‐ Supporting information 9 Table. Survival of hookworm L3, free‐living adults, and L3 or growth stages of *S. stercoralis* and *Rhabditis* sp. after exposure to 10% formalin compared to DW.

**Supporting Information 10** TableS10 ‐ Supporting information 10 Table. Treatment outcome of a single or consecutive three doses of 400 mg albendazole in the treatment of *Rhabditis* sp. alone and co‐infected with hookworm and/or *S. stercoralis* positive cases, 21 days after albendazole administration using agar plate culture.

**Supporting Information 11** TableS11 ‐ Supporting information 11 Table. Comparative number of *Rhabditis* sp. mono‐cultured‐positive, and co‐cultured with hookworm‐, and/or *S. stercoralis*‐positive human stool cases between agar plate culture and 1:20 water‐diluted stool suspension.

**Supporting Information 12** TableS12 ‐ Supporting information 12 Table. Comparative number of *Rhabditis* sp. mono‐cultured‐positive, and co‐cultured with hookworm‐positive dog stool cases between agar plate culture and 1:20 water‐diluted stool suspension.

**Supporting Information 13** TableS13 ‐ Supporting information 13 Table. Comparative number of L3 per gram of stool of hookworm and *S. stercoralis*, and number of growing stages of *Rhabditis* sp. produced from human stools in mono‐cultured and co‐cultured conditions between agar plate culture and 1:20 water‐diluted stool suspension.

**Supporting Information 14** TableS14 ‐ Supporting information 14 Table. Comparative number of L3 per gram of stool of hookworm and number of growing stages of *Rhabditis* sp. in stray dog stools between agar plate culture and 1:20 water‐diluted stool suspension.

## Data Availability

The data that support the findings of this study are available from the corresponding author upon reasonable request.
