# Supplementary material for: Comparative Effect of Water‐Diluted Stool on the Growth and Development of Rhabditis sp., Strongyloides stercoralis, and Hookworm
Source: Vet Med Int. 2026 Oct 7;2026:3238555. doi: 10.1155/vmi/3238555 (PMC13643833; doi:10.1155/vmi/3238555)
Supplement: Supplementary file 1 [file VMI-2026-3238555-s001.docx]

**Supplementary material Table 13.** Comparative number of L3 per gram of stool of hookworm and *S. stercoralis*, and number of growing stages of *Rhabditis* sp. produced from human stools in mono-cultured and co-cultured conditions between agar plate culture and 1:20 water-diluted stool suspension.

|  | **No. of L3 or growing stages per gram of stool**  **Median [IQR (Q1-Q3)]** |  | **P** |
| --- | --- | --- | --- |
| **Cultured conditions** | **1:20 water-diluted stool suspension** | **Agar plate culture** |  |
| *Rhabditis* sp. alone (n = 6) | 0 [0 (0-0)] | 7,250 [1,000 (7,000-8,000)] | 0.003 |
| *Rhabditis* sp. + hookworm (n = 5) | 0 [75 (0-75)] | 9,000 [97,250 (7,750-105,000)] | 0.007 |
| *Rhabditis* sp. + *S. stercoralis* (n = 4) | 0 [1,400 (0-1,400)] | 9,500 [8,250 (6,750-15,000)] | 0.018 |
| *Rhabditis* sp. + hookworm and *S. stercoralis* (n = 3) | 0 [NA] | 9,500 [NA] | 0.046 |
| Overall *Rhabditis* sp. (n = 18) | 0 [0 (0-0)] | 8,500 [11,000 (7,000-18,000)] | <0.001 |
| Hookworm + *Rhabditis* sp. (n = 5) | 340 [441 (201-642)] | 310 [385 (185-570)] | 0.602 |
| Hookworm + *Rhabditis* sp. and *S. stercoralis* (n = 3) | 86 [NA] | 78 [NA] | 0.513 |
| Overall hookworm (n = 8) | 201 [285 (95-380)] | 185 [259 (88-347)] | 0.674 |
| *S. stercoralis* + *Rhabditis* sp. (n = 4) | 396 [169 (326-495)] | 650 [263 (510-773)] | 0.020 |
| *S. stercoralis* + *Rhabditis* sp. and hookworm (n = 3) | 310 [NA] | 460 [NA] | 0.126 |
| Overall *S. stercoralis* (n = 7) | 342 [140 (310-450)] | 540 [300 (460-760)] | 0.021 |

NA, not applicable
