# Supplementary material for: Comparative Effect of Water‐Diluted Stool on the Growth and Development of Rhabditis sp., Strongyloides stercoralis, and Hookworm
Source: Vet Med Int. 2026 Oct 7;2026:3238555. doi: 10.1155/vmi/3238555 (PMC13643833; doi:10.1155/vmi/3238555)
Supplement: Supplementary file 2 [file VMI-2026-3238555-s002.docx]

**Supplementary material Table 12.** Comparative number of *Rhabditis* sp. mono-cultured-positive, and co-cultured with hookworm-positive dog stool cases between agar plate culture and 1:20 water-diluted stool suspension.

|  | **No. of positivity** |  |  |  | **P** |
| --- | --- | --- | --- | --- | --- |
| **Cultured conditions** | **Agar plate culture** |  | **1:20 water-diluted stool suspension** |  |  |
|  | *Rhabditis* sp. | Hookworm | *Rhabditis* sp. | Hookworm |  |
| *Rhabditis* sp. (n =15) | 15 ^a^ | NA | 3 ^a^ | NA | ^a^ <0.001 |
| *Rhabditis* sp. + hookworm (n =10) | 10 ^b^ | 10 | 4 ^b^ | 10 | ^b^ 0.011 |
| Total (n = 25) | 25 (100%) ^c^ | 10 (100%) | 7 (28%) ^c^ | 10 (100%) | ^c^ <0.001 |

NA = not applicable
