# Supplementary material for: Comparative Effect of Water‐Diluted Stool on the Growth and Development of Rhabditis sp., Strongyloides stercoralis, and Hookworm
Source: Vet Med Int. 2026 Oct 7;2026:3238555. doi: 10.1155/vmi/3238555 (PMC13643833; doi:10.1155/vmi/3238555)
Supplement: Supplementary file 3 [file VMI-2026-3238555-s003.docx]

**Supplementary material Table 11.** Comparative number of *Rhabditis* sp. mono-cultured-positive, and co-cultured with hookworm-, and/or *S. stercoralis*-positive human stool cases between agar plate culture and 1:20 water-diluted stool suspension.

|  | **No. of positivity** |  |  |  |  |  | P |
| --- | --- | --- | --- | --- | --- | --- | --- |
| **Cultured conditions** | **Agar plate culture** |  |  | **1:20 water-diluted stool suspension** |  |  |  |
|  | *Rhabditis* sp. | *S. stercoralis* | Hookworm | *Rhabditis* sp. | *S. stercoralis* | Hookworm |  |
| *Rhabditis* sp. (n=6) | 6 ^a^ | NA | NA | 1 ^a^ | NA | NA | ^a^ 0.015 |
| *Rhabditis* sp. + hookworm (n=5) | 5 ^b^ | NA | 5 | 1 ^b^ | NA | 5 | ^b^ 0.048 |
| *Rhabditis* sp. + *S. stercoralis* (n=4) | 4 ^c^ | 4 | NA | 1 ^c^ | 4 | NA | ^c^ 0.143 |
| *Rhabditis* sp. + *S. stercoralis* + hookworm (n=3) | 3 ^d^ | 3 | 3 | 1 ^d^ | 3 | 3 | ^d^ 0.400 |
| Total (n=18) | 18 (100%) ^e^ | 7 (100%) | 8 (100%) | 4 (22.2%) ^e^ | 7 (100%) | 8 (100%) | ^e^ <0.001 |

NA = Not applicable
