# Supplementary material for: Comparative Effect of Water‐Diluted Stool on the Growth and Development of Rhabditis sp., Strongyloides stercoralis, and Hookworm
Source: Vet Med Int. 2026 Oct 7;2026:3238555. doi: 10.1155/vmi/3238555 (PMC13643833; doi:10.1155/vmi/3238555)
Supplement: Supplementary file 4 [file VMI-2026-3238555-s004.docx]

**Supplementary Material 10 Table.** Treatment outcome of a single or consecutive three doses of 400 mg albendazole in the treatment of *Rhabditis* sp. alone and coinfected with hookworm and/or *S. stercoralis* positive cases, 21 days after albendazole administration using agar plate culture.

|  | **No. of APC positive** |  |  |  |
| --- | --- | --- | --- | --- |
|  | ***Rhabditis* sp.**  **alone** | ***Rhabditis* sp. colonization in positive cases** |  |  |
|  |  | ***S. stercoralis*** | **Hookworm** | ***S. stercoralis*+ Hookworm** |
| 2016 | 0 | 1 | 1 | 0 |
| 2019 | 3 | 2 | 2 | 2 |
| 2023 | 3 | 1 | 2 | 1 |
| Total | 6 | 4 | 5 | 3 |
| 21 days post-treatment | 0 | 0 | 0 | 0 |
| Cure rate | 100 | 100 | 100 | 100 |
