# Supplementary material for: Comparative Effect of Water‐Diluted Stool on the Growth and Development of Rhabditis sp., Strongyloides stercoralis, and Hookworm
Source: Vet Med Int. 2026 Oct 7;2026:3238555. doi: 10.1155/vmi/3238555 (PMC13643833; doi:10.1155/vmi/3238555)
Supplement: Supplementary file 5 [file VMI-2026-3238555-s005.docx]

**Supplementary Material 9 Table.** Survival of hookworm L3, free-living adults, and L3 or growth stages of *S. stercoralis* and *Rhabditis* sp. after exposure to 10% formalin compared to DW.

|  | **Hookworm** | ***S. stercoralis*** |  | ***Rhabditis* sp.** |  |
| --- | --- | --- | --- | --- | --- |
| **Motile worms** | **L3 (n = 30)** | **L3 (n = 30)** | **Free-living adults (n = 10)** | **Growth stages (n = 30)** | **Free-living adults (n = 10)** |
| After exposure to formalin at |  |  |  |  |  |
| 5 minutes | 30 | 2 | 0 | 1 | 0 |
| 7 minutes | 30 | 0 | 0 | 0 | 0 |
| 30 minutes | 30 | ND | ND | ND | ND |
| 45 minutes | 21 | ND | ND | ND | ND |
| In DW |  |  |  |  |  |
| 5 minutes | 30 | 30 | 8 | 30 | 10 |
| 7 minutes | 30 | 30 | 7 | 30 | 10 |
| 30 minutes | 30 | ND | ND | ND | ND |
| 45 minutes | 30 | ND | ND | ND | ND |

ND, not done.

Thirty hookworm L3, thirty *S. stercoralis* L3, ten *S. stercoralis* free-living adults, thirty Rhabditid growth stages, and ten rhabditid free-living adults were each transferred to separate 35 mm dishes containing 1.5 mL of either 10% formalin or distilled water. After 5, 7, 30, and 45 minutes of exposure, the number of viable (motile) worms was counted in both conditions.
