# Supplementary material for: Comparative Effect of Water‐Diluted Stool on the Growth and Development of Rhabditis sp., Strongyloides stercoralis, and Hookworm
Source: Vet Med Int. 2026 Oct 7;2026:3238555. doi: 10.1155/vmi/3238555 (PMC13643833; doi:10.1155/vmi/3238555)
Supplement: Supplementary file 6 [file VMI-2026-3238555-s006.docx]

**Supplementary Material Table 8.** Comparing the prevalence of hookworm infection and strongyloidiasis with *Rhabditis* sp. colonization rate before and after the 2014-2016 El Niño-induced drought

|  | **Prevalence** |  |  |
| --- | --- | --- | --- |
| **Year** | **Hookworm** | ***S. stercoralis*** | ***Rhabditis* sp.** |
| Before drought |  |  |  |
| 2008 (N=296) | 68.6 | 28.7 | 0.34 |
| 2009 (N=100) | 52 | 28 | 0 |
| 2011 (N=600) | 57 | 33 | 0 |
| 2012 (N=299) | 64.5 | 26.1 | 0 |
| Average (N=1295) | 61 | 30 | 0.08 ^a^ |
| After drought |  |  |  |
| 2016 (N=282) | 26.2 | 17.4 | 0.71 |
| 2019 (N=304) | 8.6 | 8.9 | 2.96 |
| 2023 (N=225) | 4.44 | 4.89 | 3.11 |
| Average (N=811) | 13.6 | 10.7 | 2.22 ^a^ |

^a^ P<0.001, Data on the prevalence of hookworm infection and strongyloidiasis in 2008, 2012, 2016, 2019, and 2023 were retrieved from our previous study [3].
