# Supplementary material for: Comparative Effect of Water‐Diluted Stool on the Growth and Development of Rhabditis sp., Strongyloides stercoralis, and Hookworm
Source: Vet Med Int. 2026 Oct 7;2026:3238555. doi: 10.1155/vmi/3238555 (PMC13643833; doi:10.1155/vmi/3238555)
Supplement: Supplementary file 7 [file VMI-2026-3238555-s007.docx]

**Supplementary Material 3 Table.** Rainfall and number of rainy days measured by the local meteorological station 0747.

|  | **Rainfall in mm (No. of rainy day)** | | | | | | | | | | | | |
| --- | --- | --- | --- | --- | --- | --- | --- | --- | --- | --- | --- | --- | --- |
|  | **Jan** | **Feb** | **Mar** | **Apr** | **May** | **Jun** | **Jul** | **Aug** | **Sep** | **Oct** | **Nov** | **Dec** | **Tot** |
| 2014 | ND | ND | ND | ND | *6.5*  *(1)* | *2*  *(1)* | ND | *0*  *(0)* | *9*  *(3)* | *0*  *(0)* | *8.5*  *(3)* | ND | *26*  *(8)* |
| 2015 | *59*  *(9)* | *2*  *(1)* | *1*  *(1)* | 63.5  (5) | 189  (12) | 79.5  (6) | 87  (10) | 259  (11) | 142  (8) | ND | ND | 28  (7) | 910  (70) |
| 2016 | 8  (1) | ND | ND | ND | ND | ND | 188.5  (10) | 49.5  (11) | 84  (11) | 140  (8) | 48  (9) | 43.5  (4) | 561.5  (54) |
| 2017 | 91  (6) | 179  (8) | 123  (7) | 234  (14) | 152  (14) | 125  (11) | 163  (9) | 104.5  (14) | 310.5  (18) | 168  (14) | 1253  (14) | 1093  (12) | 3996  (141) |
| 2018 | 245  (12) | 42  (5) | 51  (5) | 21  (4) | 187  (8) | 15.5  (8) | 31  (13) | 43  (16) | 182  (18) | 175  (19) | 299  (15) | 519  (13) | 1810.5  (136) |
| 14-18 |  |  |  |  |  |  |  |  |  |  |  |  | 1461  (81.8) |
| 2019 | *35*  *(9)* | *0*  *(0)* | ND | *0*  *(0)* | *0*  *(0)* | *0*  *(0)* | *0*  *(0)* | *0*  *(0)* | *0*  *(0)* | *0 (0)* | ND | ND | *35*  *(9)* |
| 2020 | ND | ND | ND | ND | ND | *0*  *(0)* | *27*  *(2)* | *0*  *(0)* | *0.5*  *(1)* | *19*  *(3)* | *39*  *(7)* | *6*  *(2)* | *91.5*  *(15)* |
| 2021 | 51  (4) | 10 (2) | 6  (1) | 123  (7) | 36  (6) | 20.5  (4) | 87  (11) | 168  (17) | 41  (5) | 3  (2) | 213  (6) | 253  (11) | 1011.5  (76) |
| 2022 | 88  (8) | 331  (12) | 132  (6) | 258  (10) | 93  (10) | ND | 13.5  (4) | 0.5  (1) | 0  (0) | 0  (0) | ND | ND | 916  (51) |
| 2023 | 301  (15) | 183  (7) | 113  (3) | 125  (7) | 188  (15) | 53  (16) | 79  (9) | 55  (8) | 224  (13) | 215  (7) | ND | ND | 1536  (100) |
| 19-23 |  |  |  |  |  |  |  |  |  |  |  |  | 718  (50.2) |

The data were retrieved from https://www.thaiwater.net/weather/rain, July 7, 2025.

ND = no data
