# Supplementary material for: Comparative Effect of Water‐Diluted Stool on the Growth and Development of Rhabditis sp., Strongyloides stercoralis, and Hookworm
Source: Vet Med Int. 2026 Oct 7;2026:3238555. doi: 10.1155/vmi/3238555 (PMC13643833; doi:10.1155/vmi/3238555)
Supplement: Supplementary file 8 [file VMI-2026-3238555-s008.docx]

**Supplementary Material 6 Table.** Maximum temperature in Nakhon Si Thammarat during 2013-2023.

|  | **Maximum temperature °C during 2013-2023** | | | | | | | | | | | | |
| --- | --- | --- | --- | --- | --- | --- | --- | --- | --- | --- | --- | --- | --- |
| **Year** | **Jan** | **Feb** | **Mar** | **Apr** | **May** | **Jun** | **Jul** | **Aug** | **Sep** | **Oct** | **Nov** | **Dec** | **Aver** |
| 2013 | 32.8 | 32.6 | 36.2 | 37.5 | 36.2 | 36.5 | 36.0 | 36.3 | 36.0 | 35.4 | 33.1 | 31.7 | 35.0 |
| 2014 | 32.5 | 32.5 | 35.5 | *37.0* | *38.0* | 36.7 | *37.2* | 36.2 | 36.0 | 34.5 | 33.6 | 33.5 | *35.3* |
| 2015 | 31.6 | 32.4 | 34.9 | *37.0* | *37.1* | *37.1* | *37.0* | 36.2 | 35.1 | 35.0 | 33.2 | 32.9 | *35.0* |
| 2016 | 33.7 | 32.5 | 34.6 | *38.0* | *38.1* | 36.5 | 35.5 | 36.0 | 36.2 | 35.2 | 33.6 | 33.5 | *35.3* |
| 2017 | 32.6 | 33.4 | 35.0 | 35.7 | 35.7 | 36.5 | 35.5 | 35.6 | 35.8 | 35.8 | 33.5 | 32.7 | **34.8** |
| 2018 | 32.5 | 33.0 | 34.1 | 35.6 | 35.2 | 36.5 | 36.0 | 36.0 | 37.0 | 34.4 | 32.2 | 34.3 | **34.7** |
| 2019 | 32.5 | 33.1 | 36.8 | *37.4* | *38.4* | *37.1* | *37.3* | *37.0* | 35.5 | 33.9 | 33.1 | 31.8 | *35.3* |
| 2020 | 32.6 | 32.7 | 36.5 | 36.3 | 35.8 | 35.1 | 34.7 | 35.2 | 35.4 | 34.5 | 32.6 | 32.3 | **34.5** |
| 2021 | 31.5 | 33.4 | 36.4 | 37.0 | 35.5 | 36.2 | 36.9 | 36.1 | 35.5 | 35.3 | 33.6 | 31.8 | **34.9** |
| 2022 | 33.0 | 33.2 | 34.8 | 36.2 | 36.5 | 35.5 | 35.0 | 36.1 | 35.5 | 33.5 | 33.5 | 32.2 | **34.6** |
| 2023 | 33.4 | 32.5 | 34.2 | 36.5 | 37.0 | 36.4 | 36.5 | 37.0 | 35.5 | 34.9 | 33.3 | ND | *35.2* |

The data were retrieved from https://www.tmd.go.th, July 1, 2025
