# Supplementary material for: Comparative Effect of Water‐Diluted Stool on the Growth and Development of Rhabditis sp., Strongyloides stercoralis, and Hookworm
Source: Vet Med Int. 2026 Oct 7;2026:3238555. doi: 10.1155/vmi/3238555 (PMC13643833; doi:10.1155/vmi/3238555)
Supplement: Supplementary file 9 [file VMI-2026-3238555-s009.docx]

**Supplementary Material 5 Table.** Rainfall in mm in Thailand, the eastern coast of southern Thailand, Nakhon Si Thammarat (NST) Province, and 2 local meteorological stations, and dry spell length (DSL) days in NST Province during 2014-2023.

| **Year** | **Rainfall** | | | | | **No. of DSL days** | |
| --- | --- | --- | --- | --- | --- | --- | --- |
|  | **Thailand** | **Southern Thailand** | **NST** | **STN 0064** ^a^ | **STN 0747** ^a^ | **NST**  **(dry season)** | **NST**  **(wet season)** |
| 2014 | 1520.4 | 1878 | 2318.5 | 107.5 | 26 | 51 | 1 |
| 2015 | 1419.7 | 1648 | 2091.5 | 1306 | 910 | 31 | 2 |
| 2016 | 1716.1 | 1911 | 2449.3 | 2179 | 561.5 | 29 | 4 |
| 2017 | 2017.1 | 2767 | 4478.4 | 4757 | 3996 | 17 | 6 |
| 2018 | 1660.9 | 1988 | 2218.8 | 1733 | 1810.5 | 11 | 5 |
| 2019 | 1343.4 | 1611 | 2132.2 | 195 | 35 | 38 | 5 |
| 2020 | 1527.3 | 2154 | 3141.6 | 216.5 | 91.5 | 11 | ND |
| 2021 | 1759.3 | 2083 | 2718 | 670 | 1011.5 | ND | ND |
| 2022 | ND | ND | 3521 | 965 | 916 | ND | ND |
| 2023 | ND | ND | 1888 | 1466 | 1536 | ND | ND |

^a^ Reference station in this study, ND = no data, STN = Local meteorological station

The data were retrieved from https://www.thaiwater.net/weather/rain, July 1,2025
