# Supplementary material for: Comparative Effect of Water‐Diluted Stool on the Growth and Development of Rhabditis sp., Strongyloides stercoralis, and Hookworm
Source: Vet Med Int. 2026 Oct 7;2026:3238555. doi: 10.1155/vmi/3238555 (PMC13643833; doi:10.1155/vmi/3238555)
Supplement: Supplementary file 10 [file VMI-2026-3238555-s010.docx]

**Supplementary Material 4 Table.** Rainfall and rainy days in Nakhon Si Thammarat during 2013-2016.

|  | **Monthly rainfall in mm (rainy days) during 2013-2016** | | | | | | | | | | | | |
| --- | --- | --- | --- | --- | --- | --- | --- | --- | --- | --- | --- | --- | --- |
| **Year** | **Jan** | **Feb** | **Mar** | **Apr** | **May** | **Jun** | **Jul** | **Aug** | **Sep** | **Oct** | **Nov** | **Dec** | **Tot** |
| 2013 | 84.7  (11) | 160.8  (11) | 5.1  (2) | 290  (9) | 276.5  (15) | 73.4  (11) | 125  (17) | 126  (14) | 88.5  (13) | 218  (24) | 1050  (21) | 342  (15) | 2840  (163) |
| 2014 | 267  (12) | *1.6*  *(2)* | *0* | *1.4*  *(1)* | 202  (13) | 94  (11) | 76  (16) | 159  (17) | 123  (21) | 337  (22) | 418  (21) | 639  (24) | 2318  (160) |
| 2015 | *69.5*  *(2)* | *18.3*  *(2)* | *0.2*  *(1)* | 112  (7) | 65  (11) | 63.7  (10) | 96.3  (14) | 215  (16) | 146  (19) | 327  (19) | 579  (24) | 399  (20) | 2091  (155) |
| 2016 | 114.7  (12) | 118.3  (10) | 0 | 0 | 44.4  (11) | 114.7  (14) | 140  (17) | 38.4  (15) | 116  (19) | 272  (23) | 379.5  (24) | 1111  (23) | 2449  (168) |

The data were retrieved from https://www.tmd.go.th, July 1, 2025
