# Supplementary material for: Comparative Effect of Water‐Diluted Stool on the Growth and Development of Rhabditis sp., Strongyloides stercoralis, and Hookworm
Source: Vet Med Int. 2026 Oct 7;2026:3238555. doi: 10.1155/vmi/3238555 (PMC13643833; doi:10.1155/vmi/3238555)
Supplement: Supplementary file 11 [file VMI-2026-3238555-s011.docx]

**Supplementary Material 2** **Table.** Rainfall and number of rainy days measured by the local meteorological station 0064.

|  | **Rainfall in mm (No. of rainy day)** | | | | | | | | | | | | |
| --- | --- | --- | --- | --- | --- | --- | --- | --- | --- | --- | --- | --- | --- |
|  | **Jan** | **Feb** | **Mar** | **Apr** | **May** | **Jun** | **Jul** | **Aug** | **Sep** | **Oct** | **Nov** | **Dec** | **Tot** |
| 2014 | ND | ND | ND | ND | *0*  *(0)* | *4*  *(3)* | *47*  *(4)* | *7*  *(4)* | *49.5*  *(3)* | *0*  *(0)* | *0*  *(0)* | ND | *107.5*  *(14)* |
| 2015 | *0*  *(0)* | *1*  *(1)* | *0*  *(0)* | 133  (8) | 370  (10) | 116  (11) | 140  (9) | 124  (17) | 10  (8) | 1  (1) | 255  (4) | 156  (8) | 1306  (77) |
| 2016 | *0*  *(0)* | *0*  *(0)* | *0*  *(0)* | *0*  *(0)* | *0*  *(0)* | ND | 217  (9) | 65  (16) | 122  (17) | 328  (17) | 257  (15) | 1190  (12) | 2179  (86) |
| 2017 | 1355  (17) | 150  (10) | 105  (8) | 369  (13) | 82  (14) | 113  (11) | 21  (15) | 42  (7) | 207  (15) | 134  (9) | 1149  (16) | 1030  (12) | 4757  (147) |
| 2018 | 117  (16) | 19.5  (5) | 17.5  (3) | 14  (6) | 3.5  (4) | 25  (23) | 50  (28) | 56  (20) | 30  (15) | 45  (26) | 571  (17) | 784  (15) | 1733  (178) |
| 14-18 |  |  |  |  |  |  |  |  |  |  |  |  | 2016.5  (100.4) |
| 2019 | *71*  *(4)* | *9.5*  *(2)* | ND | ND | *0*  *(0)* | *5.5*  *(2)* | *5*  *(1)* | *4*  *(1)* | *86*  *(4)* | *14 (1)* | ND | ND | *195*  *(15)* |
| 2020 | ND | ND | ND | ND | ND | *35*  *(2)* | *116*  *(6)* | *47.5*  *(7)* | *0.5*  *(1)* | *0*  *(0)* | *3.5*  *(4)* | *14*  *(3)* | *216.5*  *(23)* |
| 2021 | *68*  *(3)* | *0 (0)* | *0*  *(0)* | *0*  *(0)* | *0*  *(0)* | *0*  *(0)* | *0*  *(0)* | *1*  *(1)* | *0*  *(0)* | *0*  *(0)* | 405  (14) | 196  (11) | 670  (29) |
| 2022 | 82  (7) | 606  (16) | 161  (6) | 8  (6) | 20.5  (13) | ND | ND | 27  (8) | 60.5  (4) | ND | ND | ND | 965  (60) |
| 2023 | 281  (11) | 74  (5) | 77.5  (4) | 46  (5) | 95  (10) | 30  (9) | 64  (9) | 34  (10) | 88  (12) | 299  (15) | 360  (17) | 17.5  (3) | 1466  (110) |
| 19-23 |  |  |  |  |  |  |  |  |  |  |  |  | 702.5  (47.4) |

The data were retrieved from https://www.thaiwater.net/weather/rain, July 7, 2025

ND = no data
