# Supplementary material for: Comparative Effect of Water‐Diluted Stool on the Growth and Development of Rhabditis sp., Strongyloides stercoralis, and Hookworm
Source: Vet Med Int. 2026 Oct 7;2026:3238555. doi: 10.1155/vmi/3238555 (PMC13643833; doi:10.1155/vmi/3238555)
Supplement: Supplementary file 12 [file VMI-2026-3238555-s012.docx]

**Supplementary material 1 Table.** Rainfall and rainy days in Nakhon Si Thammarat during 2006-2013.

|  | **Monthly rainfall in mm (rainy days) during 2006-2016** | | | | | | | | | | | | |
| --- | --- | --- | --- | --- | --- | --- | --- | --- | --- | --- | --- | --- | --- |
| **Year** | **Jan** | **Feb** | **Mar** | **Apr** | **May** | **Jun** | **Jul** | **Aug** | **Sep** | **Oct** | **Nov** | **Dec** | **Tot** |
| 2006 | 194.3  (14) | 322.2  (14) | 48.2  (8) | 96.5  (17) | 177.3  (15) | 297.6  (19) | 38.9  (14) | 211  (15) | 224.3  (18) | 340.3  (17) | 417.8  (21) | 422.5  (21) | 2790.9  (193) |
| 2007 | 211.4  (17) | 5.7  (4) | 133.6  (6) | 173.4  (11) | 261.9  (25) | 188  (13) | 126.9  (18) | 104.2  (13) | 160.2  (16) | 315.9  (22) | 619.5  (19) | 246  (12) | 2546.7  (176) |
| 2008 | 455.8  (13) | 88.5  (9) | 97.5  (10) | 171.7  (9) | 180.1  (21) | 50.7  (14) | 89.8  (13) | 176.8  (15) | 140.6  (12) | 287.2  (20) | 1207.6  (23) | 305.1  (16) | 3251.4  (175) |
| 2009 | 217.6  (10) | 13.7  (1) | 104  (12) | 319.2  (11) | 227.9  (22) | 22.3  (9) | 117.6  (15) | 151.1  (18) | 172.1  (16) | 96.2  (17) | 486.8  (14) | 247  (14) | 2175.5  (158) |
| 2010 | 175.4  (13) | 7.1  (4) | 79.4  (6) | 22.1  (7) | 123.2  (12) | 175.4  (14) | 132  (14) | 128.9  (15) | 109.2  (22) | 272.2  (19) | 1043.3  (27) | 479.7  (19) | 2747.9  (172) |
| 2011 | 571.4  (23) | 32.8  (3) | 1543.7  (19) | 87.9  (7) | 184.9  (17) | 128.7  (14) | 86.6  (13) | 194.1  (19) | 104  (16) | 370.9  (21) | 491.7  (22) | 404.9  (22) | 4201.6  (196) |
| 2012 | 1067.1  (19) | 8.6  (4) | 143.7  (14) | 52.3  (11) | 39.9  (10) | 56.9  (8) | 56  (7) | 141.1  (9) | 133.5  (20) | 245.5  (20) | 370  (21) | 456.9  (16) | 2771.5  (159) |
| 2013 | 84.7  (11) | 160.8  (11) | 5.1  (2) | 290  (9) | 276.5  (15) | 73.4  (11) | 125.2  (17) | 125.1(14) | 88.5  (13) | 218.2  (24) | 1050.9  (21) | 342.2  (15) | 2840.6  (163) |
| 2006-2013 |  |  |  |  |  |  |  |  |  |  |  |  | 2916  (174) |

The data were retrieved from https://www.tmd.go.th, November 29, 2025
