# Supplementary material for: Comparative Effect of Water‐Diluted Stool on the Growth and Development of Rhabditis sp., Strongyloides stercoralis, and Hookworm
Source: Vet Med Int. 2026 Oct 7;2026:3238555. doi: 10.1155/vmi/3238555 (PMC13643833; doi:10.1155/vmi/3238555)
Supplement: Supplementary file 13 [file VMI-2026-3238555-s013.docx]

**Supplementary material Table 14.** Comparative number of L3 per gram of stool of hookworm and number of growing stages of *Rhabditis* sp. in stray dog stools between agar plate culture and 1:20 water-diluted stool suspension.

|  | **No. of L3 or growing stages per gram of stool**  **Median [ IQR (Q1-Q3)]** |  | **P** |
| --- | --- | --- | --- |
| **Cultured conditions** | **1:20 water-diluted stool suspension** | **Agar plate culture** |  |
| *Rhabditis* sp. alone (n = 15) | 0 [0 (0-0)] | 14,600 [91,600 (8,400-100,000)] | <0.001 |
| *Rhabditis* sp. + hookworm (n = 10) | 0 [56 (0-56)] | 10,800 [15,400 (8,600-24,000)] | <0.001 |
| Overall *Rhabditis* sp. (n = 25) | 0 [35 (0-35)] | 14,000 [41,500 (8,500-50,000)] | <0.001 |
| Hookworm + *Rhabditis* sp. (n = 10) | 140 [660 (60-720)] | 135 [768 (42-810)] | 0.821 |
