# Supplementary material for: Comparative Effect of Water‐Diluted Stool on the Growth and Development of Rhabditis sp., Strongyloides stercoralis, and Hookworm
Source: Vet Med Int. 2026 Oct 7;2026:3238555. doi: 10.1155/vmi/3238555 (PMC13643833; doi:10.1155/vmi/3238555)
Supplement: Supplementary file 14 [file VMI-2026-3238555-s014.docx]

**Supplementary Material 7 Table.** Relative humidity in Nakhon Si Thammarat during 2013-2023.

|  | **Relative humidity during 2013-2023** | | | | | | | | | | | | |
| --- | --- | --- | --- | --- | --- | --- | --- | --- | --- | --- | --- | --- | --- |
| **Year** | **Jan** | **Feb** | **Mar** | **Apr** | **May** | **Jun** | **Jul** | **Aug** | **Sep** | **Oct** | **Nov** | **Dec** | **Aver** |
| 2013 | 85 | 84 | 81 | 82 | 84 | 81 | 82 | 81 | 80 | 86 | 88 | 86 | 83 |
| 2014 | 83 | *79* | *78* | *76* | 80 | *78* | *77* | 80 | 83 | 87 | 89 | 87 | *81* |
| 2015 | 84 | 80 | *79* | 80 | *79* | 81 | 78 | 83 | 84 | 87 | 91 | 88 | *83* |
| 2016 | 85 | 83 | 80 | 77 | 80 | 81 | 84 | 80 | 81 | 86 | 90 | 90 | 83 |
| 2017 | 91 | **84** | **82** | **85** | **82** | 80 | 81 | 82 | 85 | 85 | 89 | 88 | **85** |
| 2018 | 88 | 81 | 80 | **82** | **84** | 81 | 77 | 79 | 83 | 87 | 89 | 89 | **83** |
| 2019 | 85 | 80 | *78* | *77* | *78* | *78* | *78* | *77* | 84 | 87 | 88 | 87 | *81* |
| 2020 | 83 | 82 | 77 | **81** | **83** | 84 | 48 | 82 | 84 | 87 | 89 | 88 | **84** |
| 2021 | 86 | 80 | 79 | **83** | **84** | 81 | 79 | 81 | 81 | 85 | 92 | 86 | **83** |
| 2022 | 83 | 86 | 83 | **84** | **84** | 82 | 83 | 80 | 83 | 88 | 90 | 89 | **85** |
| 2023 | 88 | 82 | 80 | 77 | 80 | 81 | 80 | 77 | 82 | 85 | 89 | ND | 82 |

The data were retrieved from https://www.tmd.go.th, July 1, 2025
